# Study of the Reaction 2-(*p*-Nitrophenyl)ethyl Bromide + OH^−^ in Dimeric Micellar Solutions

**DOI:** 10.3390/molecules16119467

**Published:** 2011-11-11

**Authors:** María del Mar Graciani, Amalia Rodríguez, Victoria I. Martín, María Luisa Moyá

**Affiliations:** Department of Physical Chemistry, University of Seville, C/ Profesor García González 1, 41012 Seville, Spain

**Keywords:** 2-(*p*-nitrophenyl)ethyl bromide, basic dehydrobromination, dimeric surfactants, morphological transitions, ion-exchange model

## Abstract

The dehydrobromination reaction 2-(*p*-nitrophenyl)ethyl bromide + OH^−^was investigated in several alkanediyl-α-ω-bis(dodecyldimethylammonium) bromide, 12-s-12,2Br^−^ (with s = 2, 3, 4, 5, 6, 8, 10, 12) micellar solutions, in the presence of NaOH 5 × 10^−3^ M. The kinetic data were quantitatively rationalized within the whole surfactant concentration range by using an equation based on the pseudophase ion-exchange model and taking the variations in the micellar ionization degree caused by the morphological transitions into account. The agreement between the theoretical and the experimental data was good in all the dimeric micellar media studied, except for the 12-2-12,2Br^−^ micellar solutions. In this case, the strong tendency to micellar growth shown by the 12-2-12,2Br^−^ micelles could be responsible for the lack of accordance. Results showed that the dimeric micelles accelerate the reaction more than two orders of magnitude as compared to water.

## 1. Introduction

Micellar solutions contain organized surfactant aggregates that have common structural features: hydrocarbon cores composed of surfactant tails; interfacial regions containing head-groups, counterions, and water and the surrounding aqueous phase or bulk region [1]. Thus, three regions of distinctly different solvation properties, polar aqueous, non polar cores and interfacial regions of intermediate polarity are present in a single homogeneous, thermodynamically stable solution. The totality of the hydrocarbon, interfacial, and aqueous regions in micellar solutions can be treated as separate reaction regions distributed throughout the solution because the distributions of all the components are in dynamic equilibrium [2]. For bimolecular reactions between lipophilic and hydrophilic reactants dissolved in micellar solutions, the hydrophilic reactant partitions primarily between the aqueous and interfacial regions and the lipophilic reactant partitions primarily between the interfacial and hydrophobic regions. For surfactants with opposite charge to that of the water-soluble reactant, an increase in the reaction rate is found [3,4,5,6]. Conversely, if the ionic reactant is of like charge to the surfactant, rate inhibition is observed [7].

Dimeric surfactants represent a new class of surfactants. They are formed by two amphiphilic moieties connected at the level of the head groups by a spacer [8,9]. The interest in such surfactants arises from their physicochemical properties that are more favorable than those of conventional surfactants, such as much lower critical micelle concentrations (cmc), better wetting, greater surface tension lowering, and unusual morphologies. These properties could make them potentially useful in many fields of application, for example, in soil remediation, enhanced oil recovery, drug entrapment and release, *etc.* [10]. At concentrations above the cmc dimeric surfactants tend to self-associate in water to form micelles whose characteristics depend on surfactant nature as well as on temperature [8,9]. Several dimeric surfactants undergo morphological transitions when surfactant concentration increases [8,9], the dimeric micelles changing shape from spherical aggregates into elongated ones. The surfactant concentration at which this morphological transition occurs is often referred to as “second cmc” (C*). The sphere-to-rod transition is followed by variations in the characteristics of the micellar aggregates which can affect the rate of reactions. In a previous work [11], the reaction methyl naphthalene-2-sulfonate + Br^−^ was investigated in several alkanediyl-α-ω-bis(dodecyldimethylammonium) bromide, 12-s-12,2Br^−^ (with s = 2, 3, 4, 5, 6, 8, 10, 12), micellar solutions. The kinetic data within the whole surfactant concentration range were quantitatively explained by using a modified pseudophase model which took into account the micellar kinetic effects caused by morphological transitions. To the authors’ knowledge, it was the first time that kinetic micellar effects on a micelle-modified reaction were quantitatively explained in a micellar reaction media where a morphological transition occurs. As an extension of this research, the dehydrobromination reaction between 2-(*p*-nitrophenyl)ethyl bromide, PEB, and OH^-^ ions was investigated in the aqueous 12-s-12,2Br^−^ (with s = 2, 3, 4, 5, 6, 8, 10, 12) dimeric micellar solutions. The rate of this process in the micellar reaction media depends on the ion-exchange equilibrium constant, K_OH/Br_, for the competition between the bromide and the hydroxide ions for the positively charged surface of the dimeric micelles and this study could show if changes in this magnitude with micellar growth have to be considered in order to rationalize the micellar kinetic effects. Besides, this process has the advantage that the equilibrium binding constants of the organic substrate to the dimeric micelles is experimentally accessible.

## 2. Results and Discussion

### 2.1. Characteristics of the Dimeric Micellar Reaction Media

**Figure 1:**
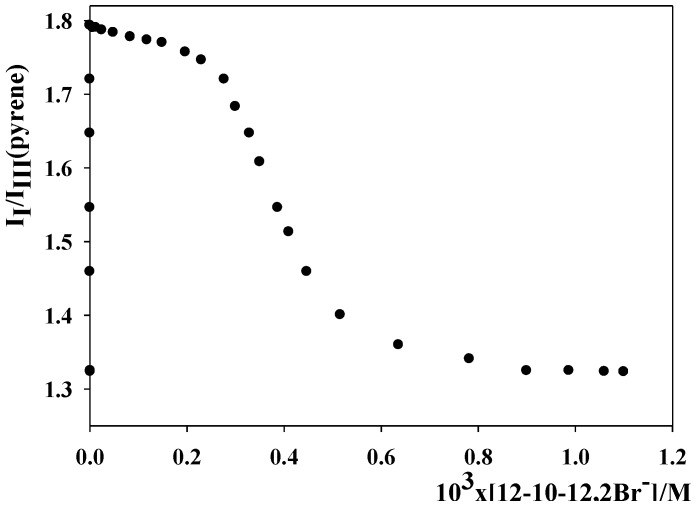
Dependence of the pyrene I_I_/I_III_ ratio on surfactant concentration in 12-10-12,2Br^−^ aqueous solutions in the presence of NaOH 5 × 10^−3^ M. T = 303 K.

Table 1 summarizes the critical micelle concentration, cmc, micellar ionization degree, α, and second cmc, C*, of the dimeric surfactant solutions in pure water at 303 K. Conductivity measurements could not be carried out in the presence of NaOH 5 × 10^−3^ M in order to determine the cmc and α values in the presence of NaOH 5 × 10^−3^ M. Since the sodium hydroxide concentration was low, the authors assumed that the micellar ionization degree was the same as that in pure water. In regard to the cmc, this magnitude was obtained in the presence of sodium hydroxide by employing a fluorescent method based on the variations of the pyrene intensity ratio *I*_I_/*I*_III_ following the micellization. All *I*_I_/*I*_III_ plots show a decrease as the total surfactant concentration increases, associated with the formation of micelles (Figure 1). The estimation of the cmc was done by using the procedure proposed by Zana [12]. The authors also assumed that the second cmc, C*, was similar in the absence as in the presence of NaOH.

**Table 1:** Critical micelle concentration, cmc, in the absence and in the presence of NaOH 5 × 10^−3^ M, micellar ionization degrees, α, and second micelle concentrations, C*, for the aqueous dimeric surfactant solutions used as reaction media. T = 303 K.

Surfactant	10^3^ × cmc/M ^a^	α ^a^	C*/M ^a^	10^3^ × cmc/M ^b^ (NaOH 5 × 10^−3^ M)
12-2-12,2Br^-^	0.95	0.17	0.016	0.39
12-3-12,2Br^-^	0.99	0.22	0.036	0.37
12-4-12,2Br^-^	1.1	0.25	0.025	0.44
12-5-12,2Br^-^	1.1	0.28	0.029	0.37
12-6-12,2Br^-^	0.99	0.31	0.028	0.39
12-8-12,2Br^-^	0.88	0.40	0.031	0.26
12-10-12,2Br^-^	0.59	0.45	0.028	0.20
12-12-12,2Br^-^	0.36	0.45	0.023	0.17

^a^ Data taken from reference [11]; ^b^ This work.

### 2.2. Kinetic Results

**Scheme 1:**
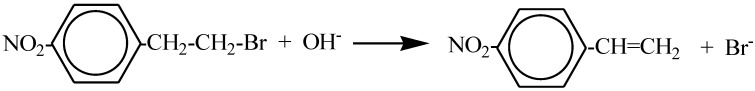
Reaction between 2-(p-nitrophenyl)ethyl bromide, PEB, and OH^−^ ions.

**Figure 2:**
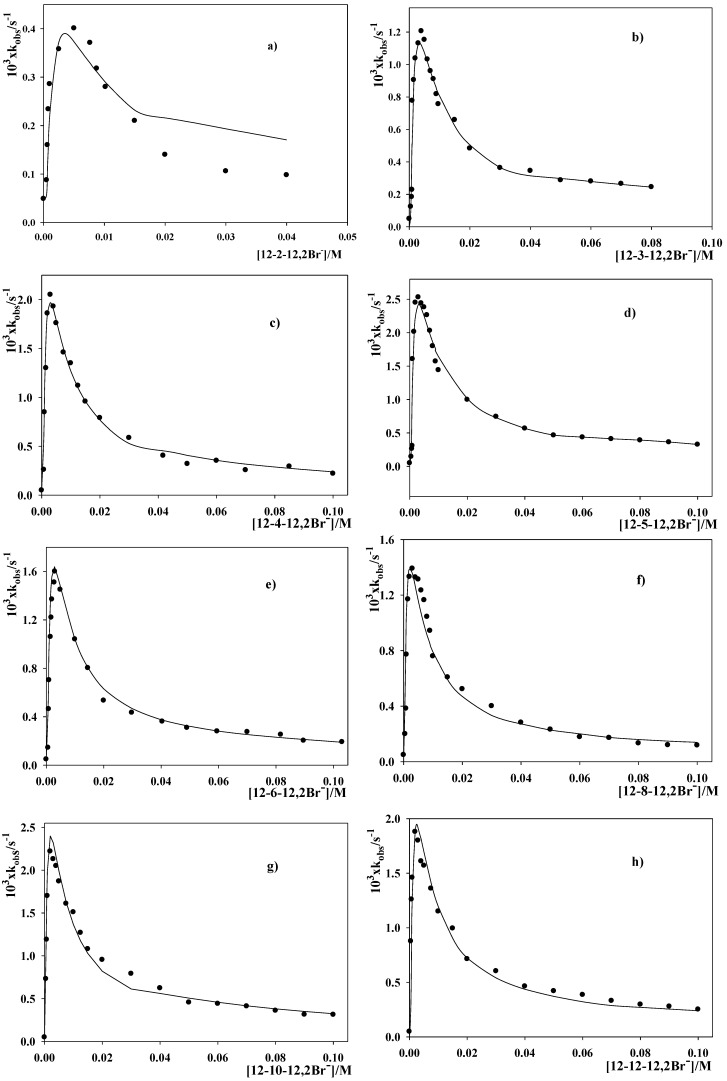
Dependence of the observed rate constant for the reaction 2-(*p*-nitrophenyl)ethyl bromide, PEB, + OH^−^ on surfactant concentration. [NaOH] = 5 × 10^−3^ M and T = 303 K. (**a**)12-2-12,2Br^−^; (**b**)12-3-12,2Br^−^; (**c**)12-4-12,2Br^−^; (**d**)12-5-12,2Br^−^; (**e**)12-6-12,2Br^−^; (**f**)12-8-12,2Br^−^; (**g**)12-10-12,2Br^−^ and (**h**)12-12-12,2Br^−^.

The dependence of the observed rate constant for the reaction between 2-(*p*-nitrophenyl)ethyl bromide, PEB, and OH^−^ (Scheme 1) on surfactant concentration in the different dimeric micellar solutions is shown in Figure 2. The hydroxide surfactant concentration was kept constant and equal to 5 × 10^−3^ M in all micellar solutions investigated.

Figure 2 shows that, in all micellar media investigated, an increase in [12-s-12,2Br^−^], at low surfactant concentration, results in a steep increase in the observed rate constant. At a well-defined [12-s-12,2Br^−^] k_obs_ reaches a maximum and a subsequent increase in surfactant concentration causes a decrease in the observed rate constant. The same behavior was found in micellar solutions of conventional cationic surfactants [12,13,14]. The existence of this maximum can be explained by considering that the reaction takes place in the aqueous as well as in the micellar pseudophases. The increment in [12-s-12,2Br^−^] at low surfactant concentration results in an acceleration because the organic substrate incorporates into the micelles and the contribution of the process occurring in the small volume of the micellar pseudophase increases (concentration effects). However, as [12-s-12,2Br^−^] increases, a diminution in the hydroxide ion concentration in the micellar pseudophase is caused by the increment of micellar aggregates present in the reaction media.

In order to rationalize the experimental kinetic data, the following expression for the observed rate constant, based on the model proposed by Quina *et al.* [15], was considered [16]:

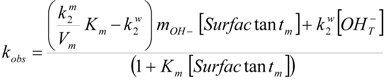
(1)

Here *w* and *m* denote the aqueous and micellar pseudophases. k_2_^w^ and k_2_^m^ are the second-order rate constants for the reaction in the aqueous and micellar pseudophases, respectively. V_m_ is the effective volume, per mole of micellized surfactant, of the region surrounding the micelle within which the ions are bound. K_m_ is the equilibrium binding constant of the organic substrate to the cationic dimeric micelles and m_OH−_ is the concentration of hydroxide ions in the micellar pseudophase per mole of micellized surfactant, m_OH−_ = [OH_m_^−^]/[Surfactant_m_]. [Surfactant_m_] is the micellized surfactant concentration, equal to the total surfactant concentration minus the cmc. [OH_T_^−^] is the total hydroxide ion concentration. (k_m_^2^/V_m_) = k_2m_ (s^−1^) is the second-order rate constant in the micellar pseudophase written with the concentrations expressed as molar ratios.

K_m_ could not be experimentally determined in the presence of NaOH and, therefore, it has to be considered as an adjustable parameter in Equation (1). However, since the sodium hydroxide concentration in the reaction media is low, no large changes for this magnitude would be expected as compared to its value in pure water. Besides, the estimation of the experimental K_m_ values will allow one to check the reliability of the K_m_ adjustable parameters obtained from the fittings of the kinetic data by using Equation (1). With this in mind, K_m_ was estimated in the aqueous dimeric micellar solutions, in the absence of NaOH, by using a spectroscopic method [17]. K_m_ can be written as:

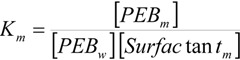
(2)
where the subscripts *w* and *m* denote the aqueous and micellar pseudophases, respectively, and [Surfactant_m_] has the same meaning as ion Equation (1). Assuming that Beer’s law is obeyed, one can write [17]:

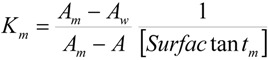
(3)
where A is the observed absorbance and A_w_ and A_m_ are the absorbances in water and of the fully micellar-bound organic substrate. In the case of 2-(*p*-nitrophenyl)ethyl bromide a high surfactant concentration would be necessary in order to measure A_m_ directly. The same result was found by Wilk *et al.* in conventional cationic micellar solutions [18,19,20]. To estimate K_m_ without the measurement of A_m_ the following equation was considered [21]:

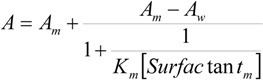
(4)

**Figure 3:**
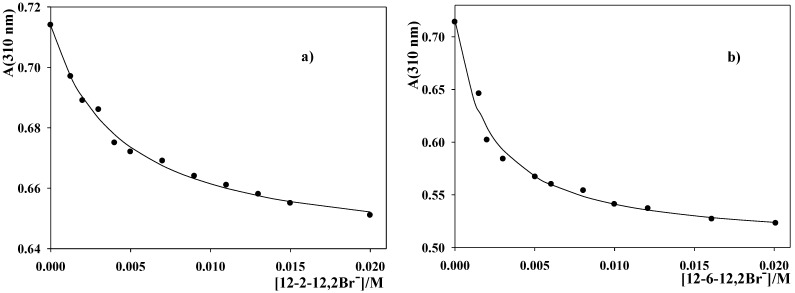
Dependence of the absorbance of 2-(*p*-nitrophenyl)ethyl bromide micellar solutions on surfactant concentration in: (**a**)12-2-12,2Br^−^ and (**b**)12-6-12,2Br^−^. The fittings were done by using Equation (4). T = 303 K.

The experimentally accessible terms of Equation (4) are A, A_w_, and [Surfactant_m_]. Figure 3 shows the fit of Equation (4) to the experimental absorbance data obtained in aqueous 12-2-12,2Br^−^ and 12-6-12,2Br^−^ micellar solutions. These data were registered at 310 nm, the wavelength at which the largest change in absorbance (by changing the surfactant concentration present in the aqueous micellar solution) was found. Nonetheless, it was verified that the value of the equilibrium binding constant obtained was independent of the wavelength chosen. The K_m_values are listed in Table 2 in parenthesis.

**Table 2:** Values of the adjustable parameters obtained from the fittings of the experimental kinetic data corresponding to the reaction 2-(*p*-nitrophenyl)ethyl bromide, PEB, + Br^−^ in several dimeric micellar solutions by using Equation (1). T = 303 K.

Surfactant	10^2^ × k_2m_ = (k_2_^m^/V_m_)/s^−1^	K_m_/M^−1^
12-2-12,2Br^−^	1.3 ± 0.3	240 ± 70 (190 ± 20)
12-3-12,2Br^−^	1.3 ± 0.1	280 ± 40 (250 ± 30)
12-4-12,2Br^−^	1.9 ± 0.1	380 ± 50 (330 ± 30)
12-5-12,2Br^−^	2.2 ± 0.2	310 ± 50 (270 ± 30)
12-6-12,2Br^−^	1.7 ± 0.1	350 ± 50 (310 ± 30)
12-8-12,2Br^−^	1.7 ± 0.1	320 ± 40 (300 ± 40)
12-10-12,2Br^−^	1.9 ± 0.1	270 ± 50 (280 ± 30)
12-12-12,2Br^−^	2.2 ± 0.1	300 ± 50 (340 ± 30)

Values in parenthesis are the equilibrium binding constants obtained by using a spectroscopic method.

With the scope of calculating m_OH−_ for the different surfactant concentrations in the micellar reaction media, the following equations were taken into account:

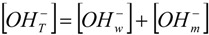
(5)

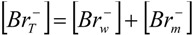
(6)

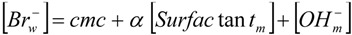
(7)

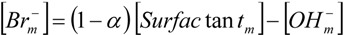
(8)

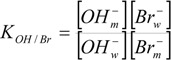
(9)
where K_OH/Br_ is the ion-exchange constant between hydroxide and bromide ions, α is the micellar ionization degree, and cmc is the critical micelle concentration. Concentrations were referred to the total solution volume. The experimental cmc values were taken from Table 1. In regard to the micellar ionization degree, it is necessary to take into account that α remains constant within the surfactant concentration range cmc < [surfactant] < C* (these α values are listed in Table 1). However, micellar growth is accompanied by a decrease in the micellar ionization degree and, consequently, α varies upon changing surfactant concentration within the range [surfactant] > C* [22,23]. The dependence of α on surfactant concentration was experimentally estimated for all the dimeric micellar media, as in previous works [11,24], by using Kuwamoto’s method [23]. Values of K_OH/Br_ for the dimeric micellar solutions were not found in the literature. The authors assumed that K_OH/Br_ for the dimeric micellar solutions investigated was similar to that corresponding to conventional alkyltrimethylammonium bromide surfactants. With this in mind, a value of 0.098 for the ion-exchange equilibrium constant was taken into account [25]. k_2_^w^ was experimentally obtained, its value being equal to 9.8 × 10^−3^ M^−1^ s^−1^ at 303 K.

Solid lines in Figure 1 show the result of fitting the kinetic data by using Equation 1. One can see that the agreement between the theoretical and the experimental data was reasonably good, with the exception of the 12-2-12,2Br^−^ micellar solutions. The lack of agreement found for the 12-2-12,2Br^−^ micellar solutions could be caused by changes in K_OH/Br_ upon changing surfactant concentration within the range [surfactant] > C* due to the strong micellar growth. All the dimeric micellar solutions investigated undergo a morphological transition upon increasing surfactant concentration [11]. However, the tendency to micellar growth depends on the spacer length [26]. The strong tendency to micellar growth shown by the dimeric surfactant with s = 2 was examined by using rheology measurements [27,28]. The viscoelastic behaviour found for 12-2-12,2Br^−^ micellar solutions was attributed to the entanglement of long and flexible aggregates. The fact that for s > 2 no viscoelastic behaviour is found in pure water points out that for s = 2 the tendency to micellar growth is stronger than for s > 2. This conclusion is also in agreement with cryogenic electronic transmission microscopy, CryoTEM, measurements carried out in 12-s-12,2Br^−^ micellar solutions by increasing surfactant concentration [26,29]. Micellar growth could also affect reactivity through changes in K_m_ and in k_2m_. However, with the exception of 12-2-12,2Br^−^ micellar solutions, the variations caused by micellar growth on these two magnitudes are small or they operate on reactivity in opposing ways since Equation 1 was adequate for fitting the kinetic data.

The values of the k_2m_ and K_m_ adjustable parameters obtained for the different dimeric micellar solutions are listed in Table 2. It is interesting to note that, within experimental errors, the K_m_ values estimated from the spectroscopic method (in parenthesis) and those obtained from the fittings are in reasonably good agreement. K_m_(spectroscopic) is always somewhat smaller than K_m_(theoretical). However, an increase in the equilibrium binding constant upon increasing the ionic strength of the medium is expected, in agreement with the results found by Wilk for PEB molecules in cetyltrimethylammonium bromide micellar solutions in the absence and in the presence of NaBr [18]. The agreement between K_m_(spectroscopic) and K_m_(theoretical) gives reliability to the fittings and seems to support the assumptions made by the authors. Besides, the reasonably good fittings shown in Figure 2 can be taken as indicative that that the ion-exchange constant does not vary substantially with micellar growth.

Table 2 shows that the equilibrium binding constant is similar for the different dimeric micellar solutions, with the exception of 12-2-12,2Br^−^ solutions for which K_m_ is smaller. The K_m_ values are similar to those found for conventional alkyltrimethylammonium bromide surfactants [13,14,24]. With regard to the k_2m_ values, this second-order rate constant does not show any dependence on the spacer length (Table 2). In order to get some information about the capacity of the dimeric micelles as catalysts for the reaction PEB + OH^−^ with respect to water, k^m^_2_ = k_2m_·V_m_ has to be estimated for the different micellar reaction media. V_m_ values for s = 2,3,4,5,6,8,10, and 12 were 0.56, 0.58, 0.59, 0.60, 0.63, 0.66, 0.70, and 0.73 dm^3^ mol in pre water, respectively [30]. The k^m^_2_ values calculated are within the range 7.3 × 10^−3^ mol^−1^ dm^3^ s^−1^ < k^m^_2_ < 16 × 10^−3^ mol^−1^ dm^3^ s^−1^, to be compared to 4.9 × 10^−5^ mol^−1^ dm^3^ s^−1^. That is, the reaction is much faster in dimeric micelles than in water. This acceleration can be explained considering that micelles accelerate reactions in which charge is delocalized in the transition state, as in the E_2_ process investigated in this work. Another factor affecting reactivity would be the disruption of the hydration shell of hydroxide ions in cationic micellar solutions, which would accelerate the process. An increase in the second order rate constant in conventional alkyltrimethylammonium bromide micellar solutions in respect to that in water was previously found by other authors [11,22,23].

## 3. Experimental

### 3.1. Materials

2-(*p*-Nitrophenyl)ethyl bromide was purchased from Fluka. Aqueous solutions of sodium hydroxide (Merck) were prepared, and hydroxide ion concentrations were determined by titration. Pyrene was from Aldrich and it was purified before use by methods reported in the literature [31]. The dimeric surfactants (Scheme 2) were synthesized [32] and characterized by ^1^H-NMR, ^13^C-NMR and elemental analysis (CITIUS, University of Seville), the results being in agreement with those previously reported. Water was obtained from a Millipore Milli-Q water system.

**Figure 4:**
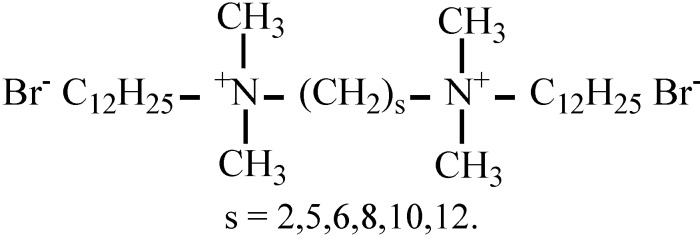
Dimeric surfactants.

### 3.2. Steady-State Fluorescence Measurements

Fluorescence measurements were done by using a Hitachi F-2500 fluorescence spectrophotometer. The temperature was kept at 303 K by a water flow cryostat connected to the cell compartment. In order to determine the cmc of the dimeric surfactant micellar solutions, 1 × 10^−6^ M pyrene surfactants solutions were prepared in twice distilled water in the presence of 5 × 10^−3^ M of NaOH and several concentrations of the dimeric surfactants, below and above the cmc. The excitation wavelength was 335 nm and the fluorescence intensities were measured at 373 nm (band 1) and 384 nm (band 3). Excitation and emission slits were 2.5 nm and a scan speed of 60 nm/min was used. The intensity ratio of the vibronic bands (1:3) is called the pyrene 1:3 ratio. Introduction of pyrene in the surfactant solutions was done as in reference [33].

### 3.3. Kinetics

Rates of dehydrobromination of 2-(*p*-nitrophenyl)ethyl bromide in the presence of hydroxide ions were determined spectrophotometrically at 318 nm. The rate measurements were performed using a Shimadzu UV-1800 and a Hitachi UV-3900 spectrophotometers. In all cases the organic substrate concentration in the reaction medium was 4 × 10^−5^ mol dm^−3^. The low solubility of 2-(*p*-nitrophenyl)- ethyl bromide in water made it necessary to prepare its solutions in acetonitrile. The percentage of acetonitrile in the reaction mixture was always 0.5 vol%. This low acetonitrile content is not expected to affect the characteristics of the aqueous solutions of the dimeric surfactants. The temperature for the kinetic runs was maintained at 303 ± 0.1 K by using a water-jacketed cell compartment.

The observed rate constant was obtained from the slopes of the ln(A_∞_ − A_t_) against time plots, with A_t_ and A_∞_ being the absorbance at time t and at the end of the reaction, respectively. The A_∞_ value was experimentally obtained by letting the reaction go to completion. Each experiment was repeated at least twice, and the observed rate constants were reproducible within a precision better than 5%. Kinetics in 12-2-12,2Br^−^ and 12-3-12,2Br^−^ could not be done for surfactant concentrations higher than 0.04 M and 0.08 M, respectively, because of solubility problems.

To test our data the observed rate constant value obtained in water at 298.2 K, k_w_ = 6.4 × 210^−3^ s^−1^ in the presence of 0.025 M of NaOH was compared to that obtained by Wilk [19], the agreement being good.

## 4. Conclusions

The dehydrobromination reaction 2-(*p*-nitrophenyl)ethyl bromide + OH^−^ was investigated in several alkanediyl-α-ω-bis(dodecyldimethylammonium) bromide, 12-s-12,2Br^−^ (with s = 2, 3, 4, 5, 6, 8, 10, 12) micellar solutions in the presence of NaOH 5 × 10^−3^ M at 303 K. In all the dimeric micellar solutions a sphere-to-rod transition takes place upon increasing surfactant concentration. The kinetic data within the whole surfactant concentration range have been quantitatively explained by considering an equation derived from the pseudophase ion-exchange model and taking the experimental decrease in the micellar ionization degree accompanying micellar growth into account. The equilibrium binding constants of the organic substrate to the dimeric micelles and the second order rate constant for the process investigated in the micellar pseudophase were obtained from the fittings. Some conclusions can be drawn for the dimeric 12-s-12,2Br^−^ micellar solutions, with s = 3,4,6,8,10,12:

- The equilibrium ion-exchange constant K_OH/Br_ for the competition between the bromide and the hydroxide ions for the positively charged surface of the dimeric micelles is similar to those for conventional alkyltrimethylammonium bromide micelles.
- K_OH/Br_ does not substantially change when the morphological transition from spherical to elongated micelles happens.
- The equilibrium binding constant of 2-(*p*-nitrophenyl)ethyl bromide molecules to the dimeric micelles is similar for all the dimeric micellar solutions. They are also similar to those found for conventional alkyltrimethylammonium bromide surfactants.
- Dimeric micelles accelerate the reaction more than two orders of magnitude as compared to pure water.

The disagreement between the theoretical and the experimental data in 12-2-12,2Br^−^ micellar solutions could be related to the strong tendency of the 12-2-12,2Br^−^ aggregates to grow. This rapid growth could cause substantial changes in the ion-exchange constant as well as in K_m_ and k_2m_.

## References

[1] Evans D.F., Wenneström H. (1994). The Colloidal Domain: Where Physics, Chemistry and Biology Meets.

[2] da Rocha Pereira R., Zanette D., Nome F. (1990). Application of the pseudophase-ion exchange model to microemulsions of anionic detergents. J. Phys. Chem..

[3] Romsted L.S., Mittal K.L. (1977). A general kinetic theory of rate enhancements for reactions between organic substrates and hydrophilic ions in micellar solutions. Micellization, Solubilization and Microemulsion.

[4] Bunton C.A., Nome F., Quina F.H., Romsted L.S. (1991). Ion binding and reactivity at charged aqueous interfaces. Acc. Chem. Res..

[5] Bunton C.A., Yao J., Romsted L.S. (1997). Micellar catalysis, a useful misnomer. Curr. Opin. Colloid Interface Sci..

[6] Savelli G., Germani R., Brinchi L., Texter J. (2001). Reactivity control by aqueous amphiphilic self-assembling systems. Reactions and Synthesis in Surfactant System.

[7] Quina F.H., Politi M.J., Cuccovia I.M., Martins-Franchetti S.M., Chaimovich H., Mittal K.L.E., Fendler J. (1982). Alkaline hydrolysis in micellar sodium dodecyl sulfate: The binding of –OH to anionic micelles. Solution Behavior of Surfactants: Theoretical and Applied Aspect.

[8] Menger F.M., Keiper J.N. (2000). Gemini surfactants. Angew. Chem. Int. Ed..

[9] Zana R.  (2002). Dimeric and oligomeric surfactants. Behavior at interfaces and in aqueous solutions: A review. Adv. Colloid Interface Sci..

[10] Zana R., Xia J. (2004). Synthesis, Interfacial and Solution-Phase Behavior and Applications.

[11] Graciani M.M., Rodríguez A., Martín V.I., Moyá M.L. (2010). Concentration and medium micellar kinetic effects caused by morphological transitions. Langmuir.

[12] Wilk K.A., Burczyk B. (1989). Micellar effects upon the reaction of hydroxide ions with 2-phenylethyl derivatives. J. Phys. Chem..

[13] Brinchi L., Germani R., Savelli G., Bunton C.A. (1999). Elimination in sulfobetaine micelles. Effect of head group bulk. J. Phys. Org. Chem..

[14] Rodríguez A., Muñoz M., Graciani M.M., Moyá M.L. (2002). Kinetic micellar effects in tetradecyltrimethylammonium bromide-pentanol micellar solutions. J. Colloid Interface Sci..

[15] Quina F.H., Chaimovich H. (1979). Ion Exchange in micellar solutions. 1. Conceptual framework for ion exchange in micellar solutions. J. Phys. Chem..

[16] Bertoncini C.R.A., Nome F., Cerichelli G., Bunton C.A. (1990). Effects of 1-butanol upon S_N_2 reactions in cationic micelles. A quantitative treatment. J. Phys. Chem..

[17] Sepúlveda L. (1974). Absorbances of solutions of cationic micelles and organic anions. J. Colloid Interface Sci..

[18] Wilk K.A. (1989). Salt effects on basic dehydrobromination reactions in nonfunctional micelles. J. Phys. Chem..

[19] Wilk K.A. (1990). Influence of *N*-hexadecyl-*N,N,N*-trimethyalmmonium nitrate on the dehydrobromination reaction of para-phenylethyl derivatives. Int. J. Chem. Kinet..

[20] Wilk K.A. (1991). Dehydrobromination reactions of para-substituted 2-phenylethyl derivatives in functional micelles. J. Phys. Chem..

[21] Novaki L.P., El Seoud O. (1999). Solvatochromism in aqueous micellar solutions: Effects of the molecular structures of solvatochromic probes and surfactants. Phys. Chem. Chem. Phys..

[22] Kuwamoto K., Asakawa T., Ohta A., Miyagishi S. (2005). Degree of micelle ionization and micellar growth for Gemini surfactants detected by 6-methoxy *N*-(3-sulfopropylquinolinium) fluorescence quenching. Langmuir.

[23] Asakawa T., Kitano H., Ohta A., Miyagishi S. (2001). Convenient estimation for counterion dissociation of cationic micelles using chloride-sensitive fluorescent probe. J. Colloid Interface Sci..

[24] Graciani M.M., Rodríguez A., Moyá M.L. (2008). Study of the reaction metil 4-nitrobenzenesulfonate and bromide ions in mixed single-chain-gemini mixed micellar solutions: Kinetic evidence for morphological transitions. J. Colloid Interface Sci..

[25] Gerakis A.M., Koupparis M.A. (1994). Physicochemical studies of the cetyltrimethylammonium bromide by using a bromide selective electrode. Talanta.

[26] Danino D., Talmon Y., Levy H., Beinert G., Zana R. (1995). Branched threadlike micelles in an aqueous solution of a trimeric surfactant. Science.

[27] Rodríguez A., Graciani M.M., Cordobés F., Moyá M.L. (2009). Water-ethylene glycol cationic dimeric micellar solutions: Aggregation, micellar growth and characteristics as reaction media. J. Phys. Chem. B.

[28] Oda R., Panizza P., Schmitz M., Lequeux F. (1997). Direct evidence of the shear-induced structure of wormlike micelles: Gemini surfactant 12-2-12. Langmuir.

[29] Berheim-Groswasser A., Zana R., Talmon Y. (2000). Sphere-to-cylinder transitions in aqueous micellar solutions of a dimeric (gemini) surfactant. J. Phys. Chem. B.

[30] Wetting S.S., Verral R.E. (2001). Thermodynamic studies of aqueos m-s-m gemini surfactant systems. J. Colloid Interface Sci..

[31] Kalyanasundaram K., Thomas J.K. (1977). Environmental effects on vibronic band intensities in pyrene monomer fluorescence and their application in studies of micellar systems. J. Am. Chem. Soc..

[32] Menger F.M., Keiper J.S., Mbadugha B.N.A., Caran K.L., Romsted L.S. (2000). Interfacial composition of Gemini surfactant micelles determined by chemical trapping. Langmuir.

[33] Zana R. (1999). Microviscosity of aqueous surfactant micelles. Effects of various parameters. J. Phys. Chem. B.

